# Increasing Oxygen Radicals and Water Temperature Select for Toxic *Microcystis* sp

**DOI:** 10.1371/journal.pone.0025569

**Published:** 2011-09-28

**Authors:** Claudia Dziallas, Hans-Peter Grossart

**Affiliations:** Department of Limnology of Stratified Lakes, Leibniz-Institute of Freshwater Ecology and Inland Fisheries, Stechlin, Germany; Argonne National Laboratory, United States of America

## Abstract

Pronounced rises in frequency of toxic cyanobacterial blooms are recently observed worldwide, particularly when temperatures increase. Different strains of cyanobacterial species vary in their potential to produce toxins but driving forces are still obscure. Our study examines effects of hydrogen peroxide on toxic and non-toxic (including a non-toxic mutant) strains of *M. aeruginosa*. Here we show that hydrogen peroxide diminishes chlorophyll a content and growth of cyanobacteria and that this reduction is significantly lower for toxic than for non-toxic strains. This indicates that microcystins protect from detrimental effects of oxygen radicals. Incubation of toxic and non-toxic strains of *M. aeruginosa* with other bacteria or without (axenic) at three temperatures (20, 26 and 32°C) reveals a shift toward toxic strains at higher temperatures. In parallel to increases in abundance of toxic (i.e. toxin gene possessing) strains and their actual toxin expression, concentrations of microcystins rise with temperature, when amounts of radicals are expected to be enhanced. Field samples from three continents support the influence of radicals and temperature on toxic potential of *M. aeruginosa.* Our results imply that global warming will significantly increase toxic potential and toxicity of cyanobacterial blooms which has strong implications for socio-economical assessments of global change.

## Introduction

Cyanobacteria are globally distributed in marine, brackish and freshwater habitats and many species form prominent blooms in warm water, which differ in their toxicity [1]. The frequency of these blooms has risen in recent decades [2], [3] and is expected to increase further, particularly due to global warming [4]. Blooms of the freshwater cyanobacterium *Microcystis aeruginosa* (Kützing) Lemmermann often produce the hepatotoxin microcystin, which endangers human and animal health. Toxic potentials (i.e. ratios of toxic vs. non-toxic cells) are highly variable in field samples [5], [6], but factors favouring growth of toxic over non-toxic strains are still unidentified. Additionally, the ecological role of microcystins has yet to be clarified [7]. However, the key for evolvement of these toxins must have been present long ago but should continue at present besides functional changings of microcystins.

High radical concentrations on the early earth and evolution of oxygenic photosynthesis by cyanobacteria and respiratory activity resulted in an increase in intra- and extracellular oxygen radicals (e.g. hydrogen peroxide) [8]. Therefore, mechanisms to reduce detrimental effects on cyanobacterial physiology, e.g. oxidation of pigments and proteins, were adventageous. Here, we hypothesize that microcystins as well as other toxins with comparable chemical structures (e.g. nodularins) function as radical scavengers. Recently, Zilliges and colleagues [9] proposed a mechanism on how microcystins are beneficial for toxin-producing cells exposed to oxidative stress. The microcystin can bind to some phycobilins and thus, protects them from degradation by reactive oxygen species. Therefore, the goal of this study is to examine the ecological consequences and to generalize our findings by testing other toxic as well as non-toxic strains.

Besides oxygen radicals, interaction with associated heterotrophic microorganisms is another important environmental factor for cyanobacteria. Toxic potential and expressed toxicity (fraction of toxin producing cyanobacteria) might potentially be strongly modified by associated heterotrophic bacteria [10], [11]. Bacteria degrading microcystins [12], [13] or inhibiting cyanobacterial growth [14] have been found on cyanobacteria *in situ* and in cultures, which would directly benefit from increasing production of secondary metabolites – such as microcystins. In addition, associated bacteria have the potential to alter environmental conditions in the vicinity of cyanobacteria by degrading, respiring and exuding organic compounds. Thus, the function of associated bacteria can be twofold: (i) protection from radicals and (ii) stimulation of toxin production via metabolism. As a result, bacterial interaction with cyanobacteria may weaken or strengthen environmental effects on cyanobacterial toxic potential and expressed toxicity and, thus, are of great relevance for predictions of global warming effects.

## Results and Discussion

### Beneficial effects of microcystins in the presence of hydrogen peroxide

**Figure 1:**
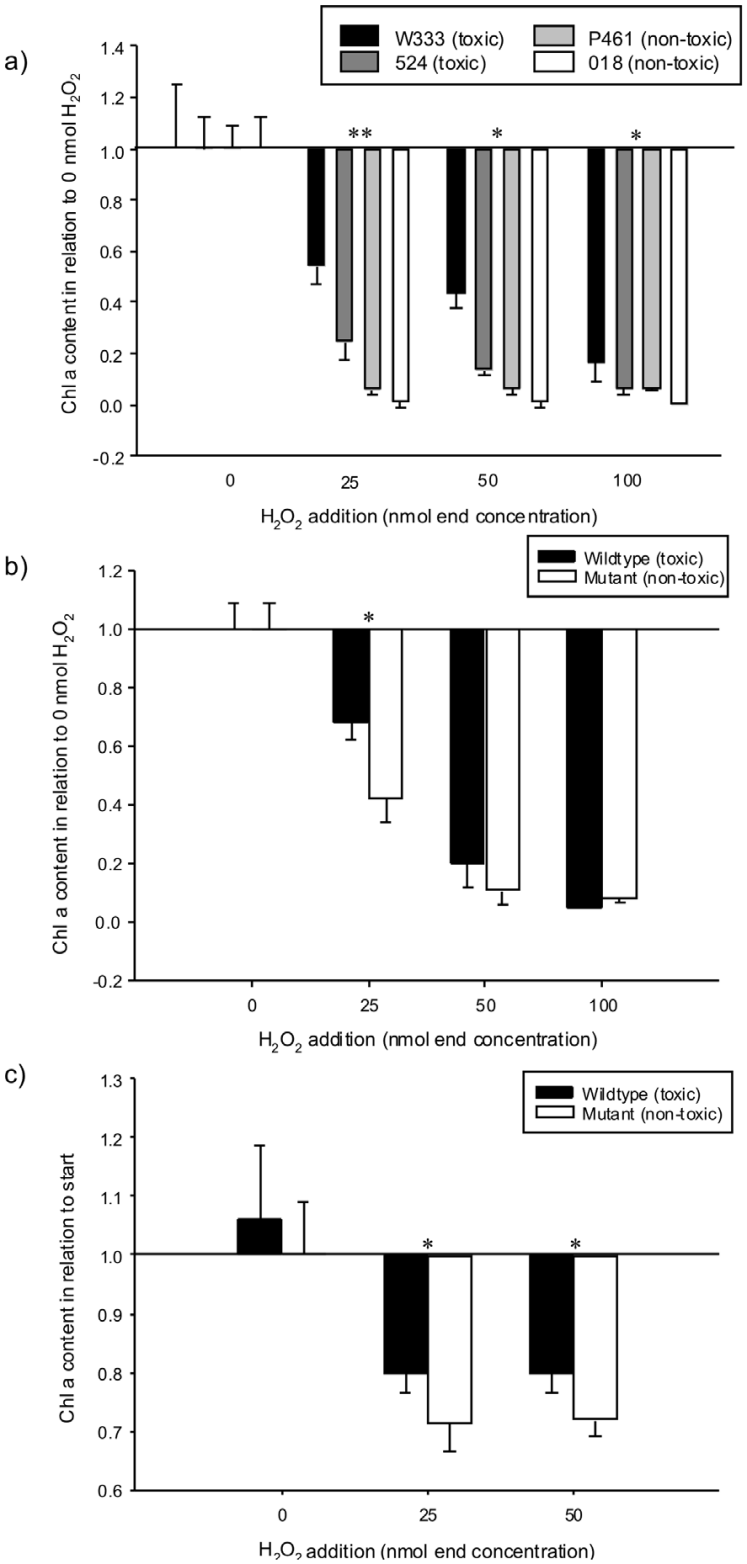
Chlorophyll a content of *M. aeruginosa* cultures after incubation with H_2_O_2_. a) after 4 days (daily H_2_O_2_ addition) for 2 toxic and 2 non-toxic strains (axenic), b) after 4 days (daily H_2_O_2_ addition) for wildtype PCC 7806 and its non-toxic mutant (xenic), c) 8 hours after H_2_O_2_ addition for toxic wildtype PCC 7806 and its non-toxic mutant (axenic plus *Pseudonocardia* sp.). Statistical significance (Mann-Whitney for a) and b) and T-test for c)) is given for comparison between toxic and non-toxic strains: * = p<0.05, ** = p<0.01.

To test whether microcystins reduce the detrimental effects of oxidative stress we incubated toxic and non-toxic strains of *M. aeruginosa* with daily additions of different concentrations of hydrogen peroxide (H_2_O_2_). H_2_O_2_ led to a significantly smaller reduction in chlorophyll a (chl a) content of toxic strains relative to non-toxic strains at 25, 50 and 100 nmol H_2_O_2_ after 4 days (Mann-Whitney, n = 6, p<0.05, Fig. 1). A significant difference in chl a content occurred for the toxic strain PCC 7806 and its microcystin-lacking mutant already after 8 hours (the half-life period of H_2_O_2_; Mann-Whitney, n = 6, p<0.05) and after 4 days of incubation at a H_2_O_2_ concentration of 25nmol (Mann-Whitney, n = 6, p = 0.014). Upon addition of H_2_O_2_ cell numbers decreased significantly in the non-toxic mutant compared to the toxic wildtype after 4 days (T-test, n = 6 each, p<0.01, Fig. S1). Microcystin content in toxic cultures declined significantly in comparison to no addition of H_2_O_2_ (T-test, n = 6 each, p<0.05, Fig. S2) probably due to chemical changes of the toxin after radical oxidation. This decline was significantly stronger in axenic than xenic cultures (T-test, n = 6 each, p<0.05, Fig. S2) indicating a contribution of heterotrophic bacteria to radical scavenging.

### Temperature dependency of toxin production by *M. aeruginosa*

**Figure 2:**
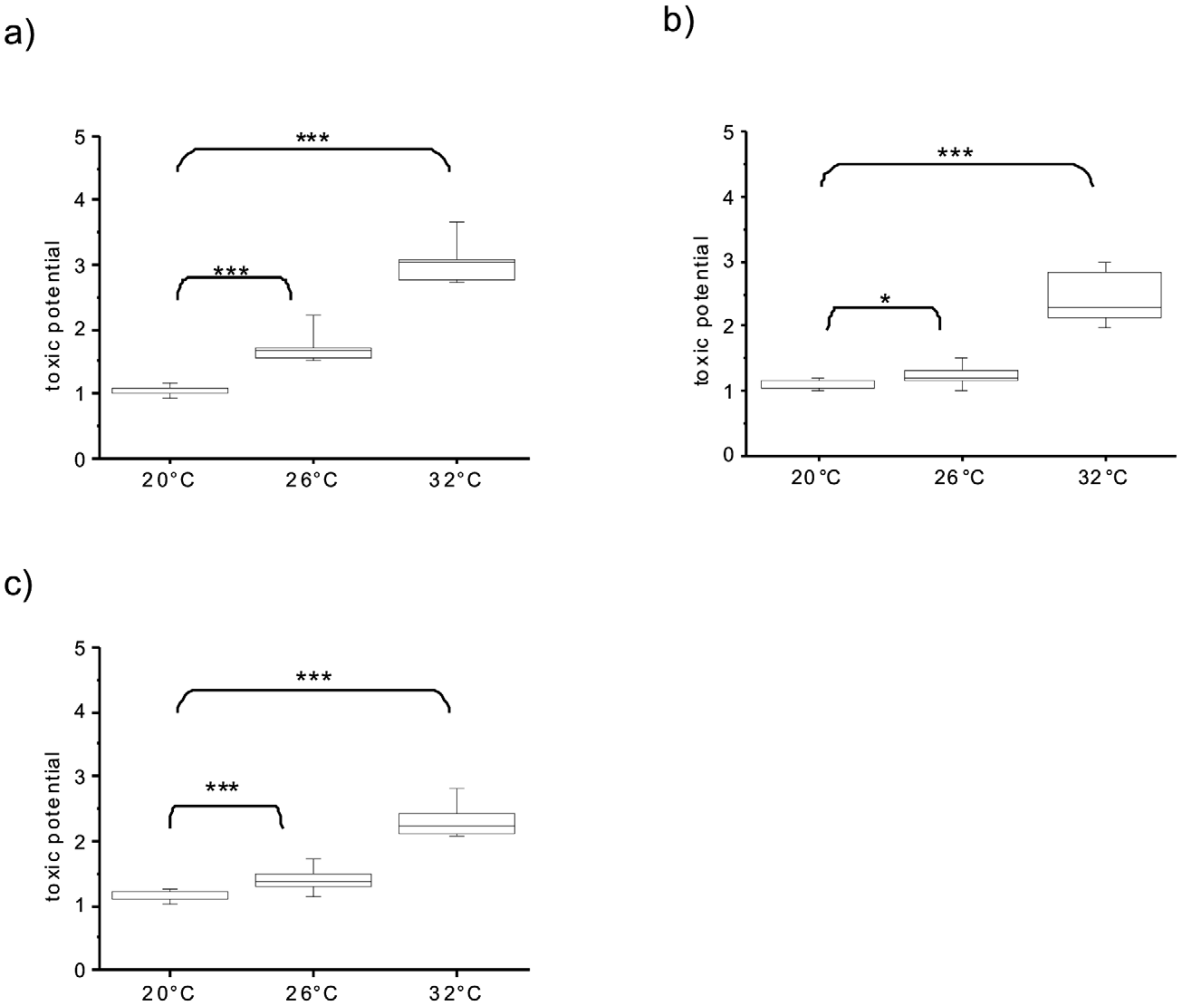
Toxic potential, i.e. ratios of toxic versus non-toxic cells of *M. aeruginosa* (medians and 25/75% quartiles are shown as boxes, range of 10/90% as whiskers). Four different strains (two toxic and two non-toxic ones) were incubated in mixed cultures for 19 days at three different temperatures a) axenic, b) and c) with inoculation of heterotrophic bacteria from a nutrient-rich lake and a nutrient-poor lake, respectively. Statistical significance (Mann-Whitney) for comparison of the samples from 26 and 32°C with 20°C: * = p<0.05, *** = p<0.001.

Since our results and the chemical structure [15] of microcystins imply a role in scavenging of free radicals, whose concentration depends on temperature, light intensity, and light quality [16], microcystins will be highly beneficial in a warmed-up future environment. Therefore, we examined temperature dependency of microcystin production by incubation of single and mixed strains of toxic and non-toxic *M. aeruginosa* at 20, 26, and 32°C – the range of present and expected summer temperatures in temperate lakes in the Mecklenburg lake district (Northeastern Germany) when considering 3.5°C higher air temperatures (IPCC 2005). To quantitatively distinguish between toxic and non-toxic and toxin-producing cells, we modified the RING-FISH method [17] targeting a microcystin-synthetase gene (toxic potential). Toxic axenic strains grew to a significant higher cell number in comparison to non-toxic strains at 26°C and particularly at 32°C than at 20°C during 19 days of incubation (Fig. 2a). This indicates a higher genetic toxic potential at elevated temperatures (Mann-Whitney, n = 24, p<0.001 pooled for both temperatures). Toxic and non-toxic axenic strains in single culture, however, did not significantly differ in growth (Kruskal-Wallis, n = 36, p>0.05 for all treatments), suggesting an inhibition of non-toxic by toxic strains in mixed axenic cultures.

Since toxin production of *M. aeruginosa* may also be modified by heterotrophic bacteria [10], we tested the effect of temperature on the growth of toxic vs. non-toxic *M. aeruginosa* strains in an experimental block design with temperature (20, 26, and 32°C) and bacteria (axenic, two xenic) as treatments. Similar to axenic cultures, toxic strains in xenic cultures grew to a significant cell number in comparison to non-toxic strains at 26°C and particularly at 32°C than at 20°C during 19 days of incubation (Fig. 2b+c). However, effects of temperature on median toxic potential of *M. aeruginosa* cultures were highest in axenic cultures (Mann-Whitney, n = 9, p<0.001 at 26°C; p = 0.001 at 32°C), intermediate with bacteria from a nutrient-poor lake (Mann-Whitney, n = 30, p<0.001 for 26 and 32°C) and smallest with bacteria from a nutrient-rich lake (Mann-Whitney, n = 30, p = 0.012 for 26°C; p<0.001 for 32°C). This strengthens the assumption that different heterotrophic bacterial communities differentially affect cyanobacterial growth [14]. Moreover, at the end of incubation the bacterial community differed with temperature but not between toxic and non-toxic strains [18].

In order to corroborate our laboratory results, we sampled *M. aeruginosa* blooms from lakes on three continents in different climatic zones. In these field samples the toxic potential of *M. aeruginosa* greatly increased with water temperature and light intensity with ratios of toxic vs. non-toxic cells ranging from 0.09 in Boulder Lake (USA, 4°C water temperature), 0.45 in Hamburg Innenalster (Germany, 22°C water temperature) to 2.67 in Lake Taihu (China, >30°C water temperature). This is consistent with our lab results.

### Temperature dependency of expressed toxicity

**Figure 3:**
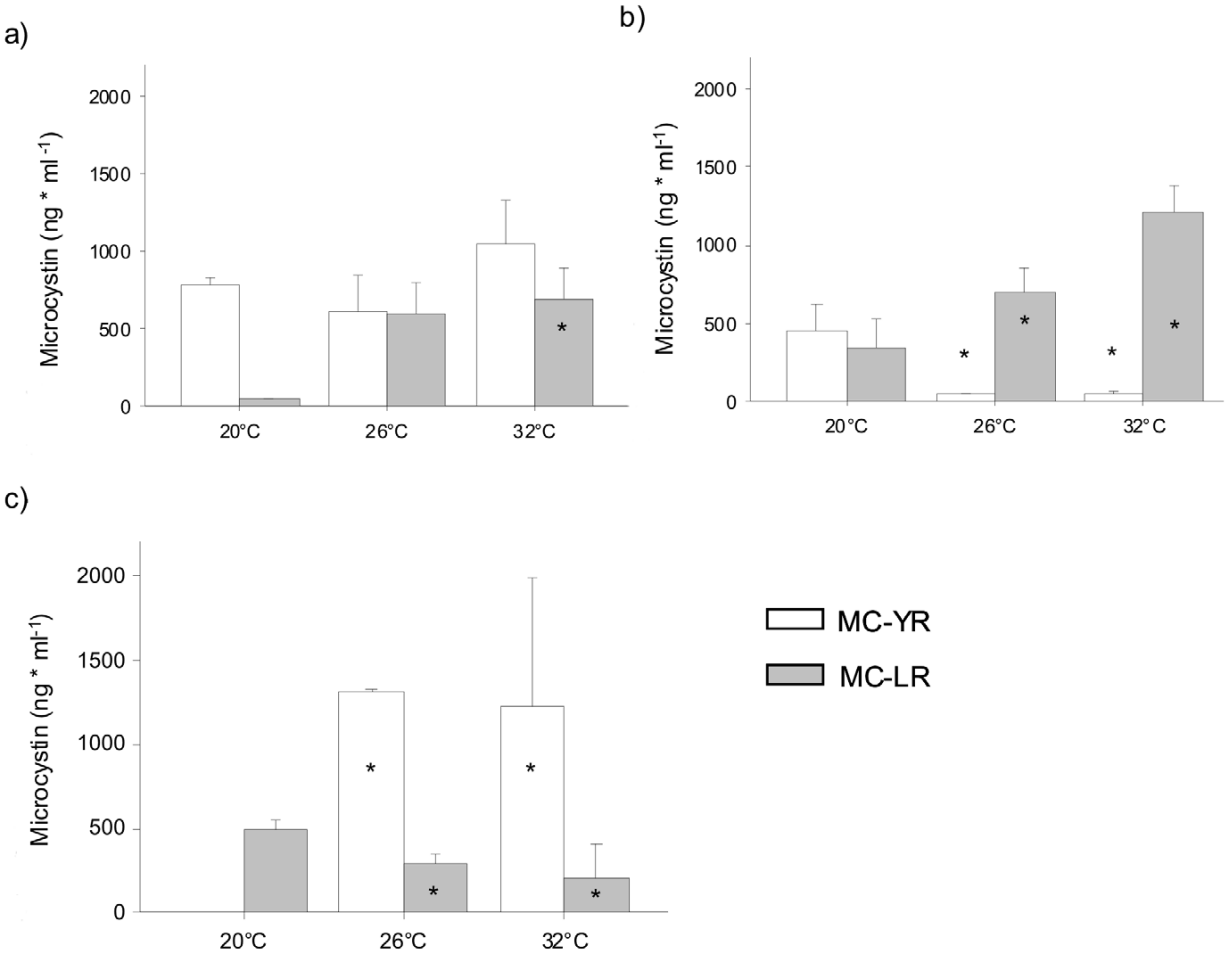
Microcystin concentration of *M. aeruginosa* in dependence of incubation temperature. Microcystin concentration per ml (medians with standard deviations) was measured at 20, 26 and 32°C of the axenic toxic strain HUB W333 a) without heterotrophic bacteria, b) with natural heterotrophic lake bacteria, c) with the non-toxic, axenic strain HUB 018. Statistical significance (Mann-Whitney) of the samples from 26 and 32°C with 20°C: * = p<0.05. Some of the data are pooled – for further information see text.

Toxic potential of *M. aeruginosa* is not necessarily predictive for actual toxin production since regulation of the respective toxin genes must be studied in an environmental context. Therefore, we adapted the RING-FISH method [17] to also detect the transcript (expressed toxicity) of the microcystin synthetase. The expressed toxicity (i.e. fraction of toxin-producing cyanobacteria) was significantly lower at 32°C than at 20 and 26°C in axenic cultures as well as in all two bacterial treatments (Mann-Whitney, n = 9 per treatment, p<0.05; Table 1). Even though the fraction of toxin-producing *M. aeruginosa* decreases with temperature, the amount of produced microcystin was higher at 26 and 32°C than at 20°C (Mann-Whitney, n = 9 per treatment, p≤0.05, Fig. 3). Strikingly, at 32°C expressed toxicities were significantly higher in the presence of bacteria than in the axenic treatment (Table 1) and lowest in the axenic single strain culture (Kruskal-Wallis, n = 9 at 32°C, p = 0.039). Thus intra- and interspecies interactions potentially affect toxin production.

**Table 1:** Fraction of toxin-producing cyanobacterial cells of total (expressed toxicity) and total number of toxin producing cyanobacteria (medians ± standard deviations).

	Temperature	% toxin-producing cells	Number of toxin producing cells *10^5^ per ml
Toxic strain axenic	20°C	70.0±3.8	3.9±0.4
	26°C	66.1±2.1	4.2±1.4
	32°C	20.6±3.2	1.7 ± 0.2
Toxic strain with heterotrophic lake bacteria	20°C	70.0±1.5	2.7±0.4
	26°C	61.3±3.4	5.0±0.7
	32°C	30.9±3.4	7.2±0.9
Toxic and non-toxic strains, axenic	20°C	59.8±4.1	16 ±2
	26°C	35.4±14.9	12 ±4
	32°C	24.8±2.1	11 ±8

### Effects of temperature and bacteria on microcystin quantity and quality

Since *M. aeruginosa* strain HUB W333 produces two different microcystins which differ in acute toxicity we determined temperature-dependent concentration of both microcystin forms in the different treatments. Microcystin-YR is 1.4 [19] to 2.6 [20] times less toxic on tested mice than Microcystin–LR, thus changes in ratios between YR- and LR-form alter the actual toxicity. This was the case in our temperature treatments: In single strain axenic cultures Microcystin-LR concentrations increased significantly with rising temperatures (Mann-Whitney, n = 9, p = 0.05 for 20°C and 26 pooled with 32°C) whereas those of the less toxic Microcystin-YR [19], [20] did not change (Mann-Whitney, n = 6 each, p>0.05 for 26 and 32°C, Fig. 3A). Actual toxin concentrations increased with increasing temperatures even though total numbers and fractions of toxin-producing cells declined (Table 1). Likewise, Microcystin-LR strongly increased with temperature in cultures with heterotrophic bacteria (Kruskal-Wallis, n = 9, p = 0.039), while Microcystin-YR was higher in single strain cultures with heterotrophic bacteria at 20°C than at 26 and 32°C (pooled, Mann-Whitney, n = 9, p = 0.048, Fig. 3B). In the presence of bacteria, the Microcystin-LR form dominated at 26 and 32°C. In contrast, in mixed axenic cultures of both toxic and non-toxic strains (HUB W333 and HUB 018, respectively), the amount of the less toxic Microcystin-YR was significantly enhanced at 26 and 32°C (pooled) compared to 20°C (Mann-Whitney, n = 9, p = 0.024), while the more toxic Microcystin-LR was significantly lower at higher temperatures (pooled, Mann-Whitney, n = 9, p = 0.048, Fig. 3C). This demonstrates a strong effect of heterotrophic bacteria on quantity and quality of cyanobacterial toxins rendering *M. aeruginosa* more toxic at increased temperatures.

### Ecological consequences

Our experiments show that microcystins weaken the detrimental effect of H_2_O_2_ on *M. aeruginosa* and that toxin production is temperature-dependent. This is in consistency with the results of Zilliges and colleagues [9] who found that a non-toxic mutant is more sensitive against oxidative stress than the toxic wildtype. As warmer environments are often characterized by more radicals (e.g., due to higher solar irradiance) with a higher potential for cell damage (e.g., due to higher diffusibility), the proposed function of cyanobacterial toxins as radical scavengers becomes more important for cyanobacterial growth at elevated temperatures. In the Baltic Sea, the toxic cyanobacterium *Nodularia spumigena* is favoured over the co-occurring non-toxic *Aphanizomenon flos-aquae* at higher irradiances as reviewed by Kononen and colleagues [21], supporting our hypothesis. Further, the increase in intracellular amounts of nodularin with light intensity and temperature [22] parallels our microcystin studies. Radical scavenging by cyanobacterial toxins is also supported by the observation that in early spring - when *Microcystis* cells are migrating from the sediments into the warmer and well illuminated surface water - toxic cells have a recruitment advantage [23]. In the presence of H_2_O_2_, the xenic non-toxic mutant retained significantly more of its chl a content than all non-toxic axenic strains in our experiment. There are two possible explanations: (i) The mutant was grown together with a heterotrophic bacterial isolate to enhance its growth and in accordance to our results this bacterium could have weakened the toxic effect of H_2_O_2_ and thereby lowered chl a reduction in the non-toxic mutant compared to the non-toxic axenic strains. (ii) It is likely that even a partially intact microcystin peptide is capable of protecting chl a from radicals since this mutant is lacking just one but not all microcystin-synthetase genes.

In addition to microcystins, other compounds function as scavengers such as carotenoides. Higher amounts of these compounds may be an adaptation of non-toxic cyanobacteria to high temperatures, enhanced light and oxidative stress conditions, but may also serve as main protection for toxic cyanobacteria which use toxins as additional radical scavengers. As our results also show a protective role of associated bacteria, they may be essential to explain the persistence of non-toxic cyanobacterial strains even under conditions with increased oxygen radical stress. Additionally, cyanobacteria – toxic as well as non-toxic strains – can regulate their boyancy via gas vacuoles and, thus, prolong their staying in more suitable water layers with lower oxidative stress.

Shifts in dominance towards toxic and toxin-producing cells within cyanobacterial communities at increasing temperatures have already been postulated [16]. Temperature-dependent toxin production by cyanobacteria is supported by field investigations from China [24] and the US [6] which, however, could not conclusively separate temperature effects from other environmental factors. To the best of our knowledge, our experiments are the first to simultaneously examine effects of temperature on toxic and non-toxic strains of a single axenic cyanobacterial species on three levels: DNA, RNA and proteins.

Eukaryotic algae often grow slower in the absence of heterotrophic bacteria [25] which promote algal growth by producing growth hormones, signalling molecules, and other organic compounds [26]. Bacteria also affect the release of cyanobacterial exudates [27] including toxins. Thus, changes in bacterial communities with rising temperatures [28] may affect toxic potential and actual toxicity of cyanobacteria. Our results show that the number of toxin-producing cyanobacterial cells increased with temperature when bacteria were present (Kruskal-Wallis, n = 9, p = 0.027) but did not consistently increase in the other treatments (Kruskal-Wallis, n = 9, p>0.05, Table 1). Moreover, associated bacteria triggered the production of specific cyanobacterial toxins.

Due to global warming, water temperatures and radical concentrations will continue to rise in summer. The resultant enhanced and prolonged lake stratification [29] will favour developments of massive cyanobacterial blooms [4]. The comparison of *Microcystis sp.* blooms in lakes with different light intensities and temperatures confirms that an increase in temperature will not only enhance the extent of cyanobacterial blooms but also their toxic potential and presumably their expressed toxicity. Strikingly, while at higher temperatures growth of non-toxic strains was increased, this increase was always smaller than that of toxic strains in mixed cultures.

### Conclusion

**Figure 4:**
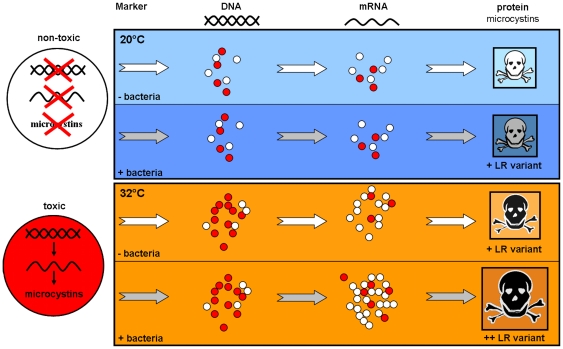
Effects of temperature and associated bacteria on toxic potential, expression and production of microcystins by *M. aeruginosa*. Red circles symbolize toxic or toxin-producing cells, white circles non-toxic ones.

Temperature and presence of oxygen radicals such as H_2_O_2_ are important environmental factors that greatly influence growth and toxic potential of cyanobacteria. Toxic potential and expressed toxicity of cyanobacterial blooms (e.g. *M. aeruginosa*) can be further increased by temperature-driven changes in associated bacterial communities (Fig. 4). There is an urgent need for further studies on combinations of abiotic and biotic factors inducing toxin production, since at higher temperatures the risk for massive toxic cyanobacterial blooms in drinking water reservoirs, cattle watering tanks, and recreational lakes will continue to increase with global warming. This is a particularly pressing issue in developing regions, where progressingly severe socio-economical problems are related to the reduced quality of surface waters brought about by eutrophication in conjunction with rising solar radiation and temperatures. A better understanding of effects of environmental factors controlling not only toxic potential but also actual expressed toxicity of various cyanobacteria should improve predictions of the extent of detrimental effects on drinking water quality and supply and should thus be of high priority to the water quality managers and researchers.

## Materials and Methods

### Strains of Microcystis aeruginosa

For the experiments we cultivated six different strains of *M. aeruginosa* (HUB 524, W333, 018 and P461; PCC 7806 and its non-toxic *mcyB*-lacking mutant) in Z-Medium [30]. HUB 524 and W333 are toxic while HUB 018 and P461 are non-toxic strains. The toxic potential was tested by PCR with primers for the microcystin-synthetase gene D (17). For removing all heterotrophic bacteria, we used the modified protocol of Domozych and colleagues [31] for isolation of exopolymer particles of green algae combined with incubation in lysozyme solution (10 g*l^−1^) for 1.5 h at 37°C. The absence of heterotrophic bacteria from the cultures was confirmed microscopically and by PCR and DGGE (see below).

### Experiment with addition of H_2_O_2_

Axenic strains of *M. aeruginosa* were incubated for four days with daily addition of H_2_O_2_ (0, 25, 50 and 100 nmol end concentration in culture) under batch conditions. Their chl a content was measured daily by extracting the chl a in 100% methanol and measuring it spectrophotometrically. The strain PCC 7806 and its non-toxic mutant were incubated with one bacterial isolate (*Pseudonocardia* sp. which was isolated from a cyanobacterial culture) to enhance their growth rate as axenic incubation resulted in no measurable increase of cell numbers during one month. For the last two strains chl a content was also measured after 3, 6, 8 and 24 hours to evaluate the acute effect of H_2_O_2_.

### Culture experiment

The axenic strains were inoculated with bacteria from the nutrient-poor Lake Stechlin or the nutrient-rich Lake Dagow for the respective treatments. The trophic level of the lakes was estimated in dependence of measured phosphate and nitrogen compounds. Both lakes are situated adjacently in northeast Germany. Lake water was filtered through 1.0 µm to remove protozoans. Bacteria (stained with SybrGold) were counted using an epifluorescence microscope and inoculated at a ratio of 1*10^6^ bacteria ml^−1^ to 1*10^4^ cyanobacteria ml^−1^. Additionally, one toxic and one non-toxic strain each were incubated jointly in mixed cultures with and without bacteria. Triplicates of all inoculated as well as axenic cyanobacterial strains were incubated in batch cultures. All incubations were done at 20°C, 26°C and 32°C in water baths and kept at 72 µE s^−1^ m^−2^ light intensity with 16 h:8 h (light : dark) periods. For RING-FISH analyses of toxic potential, samples of 5 ml were taken and filtered onto 3.0 µm Polycarbonate-filters (Nuclepore) at the start (day 0) and the end (day 19 - during the stagnation phase of cyanobacterial growth) of the experiment. For RING-FISH on mRNA, samples were directly transferred into 15 ml sterile tubes. Subsequently, the samples for DNA were frozen at −20°C and for mRNA at −80°C until processing according to the RING-FISH protocol. Samples for HPLC analysis of microcystin content were transferred into sterile 20 ml glass vials and frozen at −20°C.

### Recognition of individual genes by fluorescence *in situ* hybridization (RING-FISH)

DNA was extracted with phenol-chloroform [32]. A part of the microcystin-synthetase gene D was amplified and transcribed into an RNA-probe containing fluorescein [17]. Filters and liquid samples were hybridized with the probe and subsequently counted with an epifluorescence microscope or by using the BD FACS Aria II – flow cytometer [17]. RING-FISH was conducted for toxic cells on filters (culture experiment, field samples) and for toxin-producing cells in liquid.

### HPLC analyses of microcystin composition

Microcystin was extracted from 10 ml of cyanobacterial cultures with 75% methanol after lyophilisation [33]. Measurements of microcystin were conducted with RP-HPLC after Lawton and colleagues [34].

### Field samples

Samples were collected in sterile plastic tubes at the water surface from blooms of *Microcystis* sp. in Boulder Lake (Wisconsin, USA, October 2009), Innenalster (Northwest Germany, July 2009), and Lake Taihu (China, October 2009). Light irradiation and water temperatures at sampling time differed greatly: Boulder Lake 3.5 kWh*m^−2^*d^−1^ and 4°C, Innenalster 4 kWh*m^−2^*d^−1^ and 25°C and Lake Taihu 5 kWh*m^−2^*d^−1^ and >30°C (solar irradiation data come from Atmospheric Science Data Center). Samples were filtered onto 0.2 µm polycarbonate filters and stored frozen at −20°C.

### Statistics

Comparisons of non-normal distributed results were tested by the non-parametric Mann-Whitney-U-Test and Kruskal-Wallis-Test, normal distributed results by T-Test in SPSS 14. These tests were conducted one-tailed when appropriate (see results section). Significance level for all tests was 0.05.

## Supporting Information

Figure S1 **Growth of** ***M. aeruginosa*** **after addition of H_2_O_2_.** Growth (increase in cell numbers) in relation to experimental start of toxic *M. aeruginosa* growth strain PCC 7806 and its non-toxic mutant after four days of daily addition of H_2_O_2_. Statistical significance (T-test) for comparison of the samples from the wildtype with the mutant: ** = p<0.01, *** = p<0.001.
(TIF)

Figure S2 **Microcystin content of** ***M. aeruginosa*** **after addition of H_2_O_2_.** Microcystin content in relation to experimental start of the strain PCC 7806 without (axenic) or with (xenic) accompanying bacteria.
(TIF)
