# Supplementary figures and images for: Increasing Oxygen Radicals and Water Temperature Select for Toxic Microcystis sp
Source: PLoS One. 2011 Sep 28;6(9):e25569. doi: 10.1371/journal.pone.0025569 (PMC3182230; doi:10.1371/journal.pone.0025569)

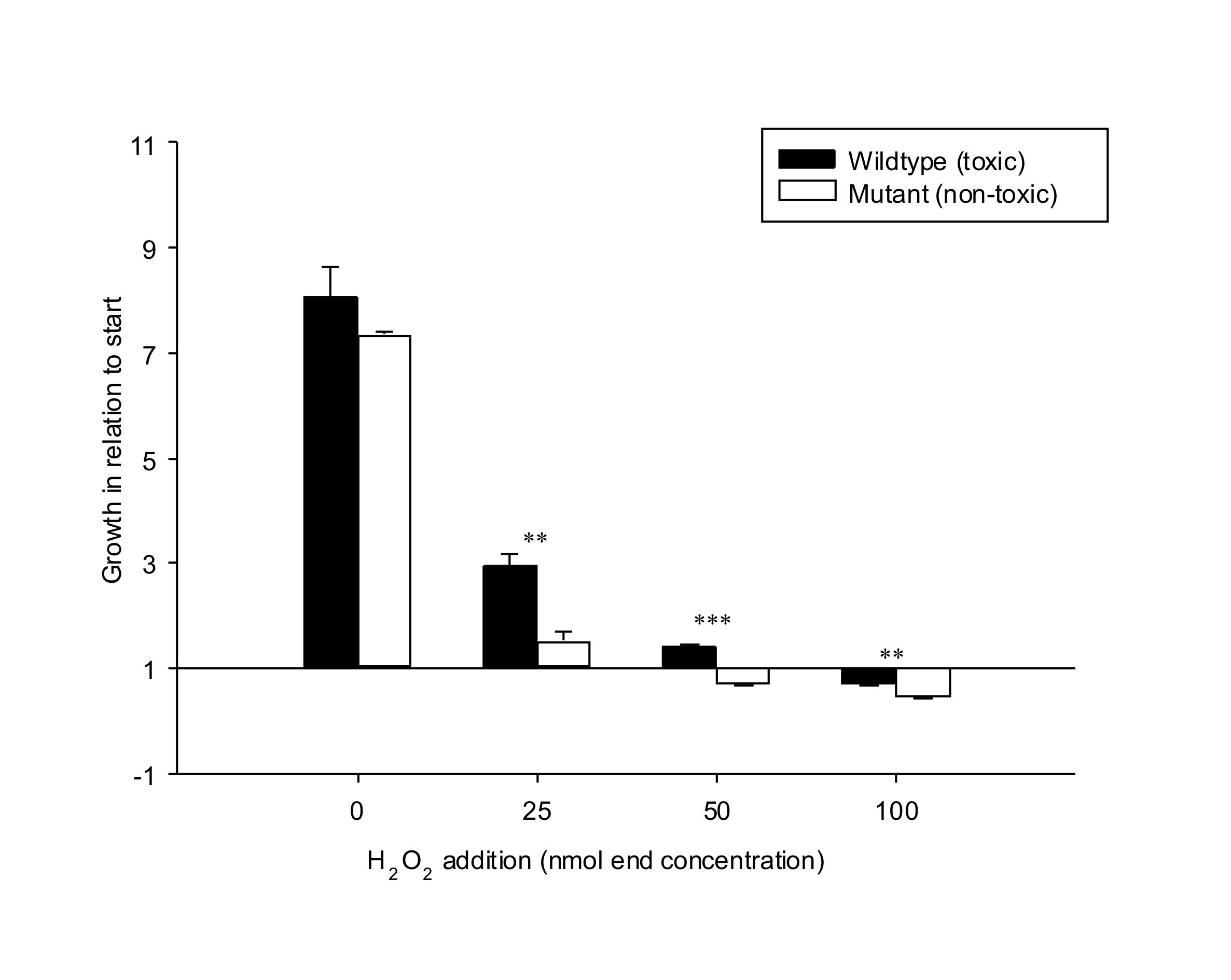

Supplement: Figure S1 — Growth of M. aeruginosa after addition of H2O2. Growth (increase in cell numbers) in relation to experimental start of toxic M. aeruginosa growth strain PCC 7806 and its non-toxic mutant after four days of daily addition of H2O2. Statistical significance (T-test) for comparison of the samples from the wildtype with the mutant: ** = p<0.01, *** = p<0.001. (TIF) [file pone.0025569.s001.tif]

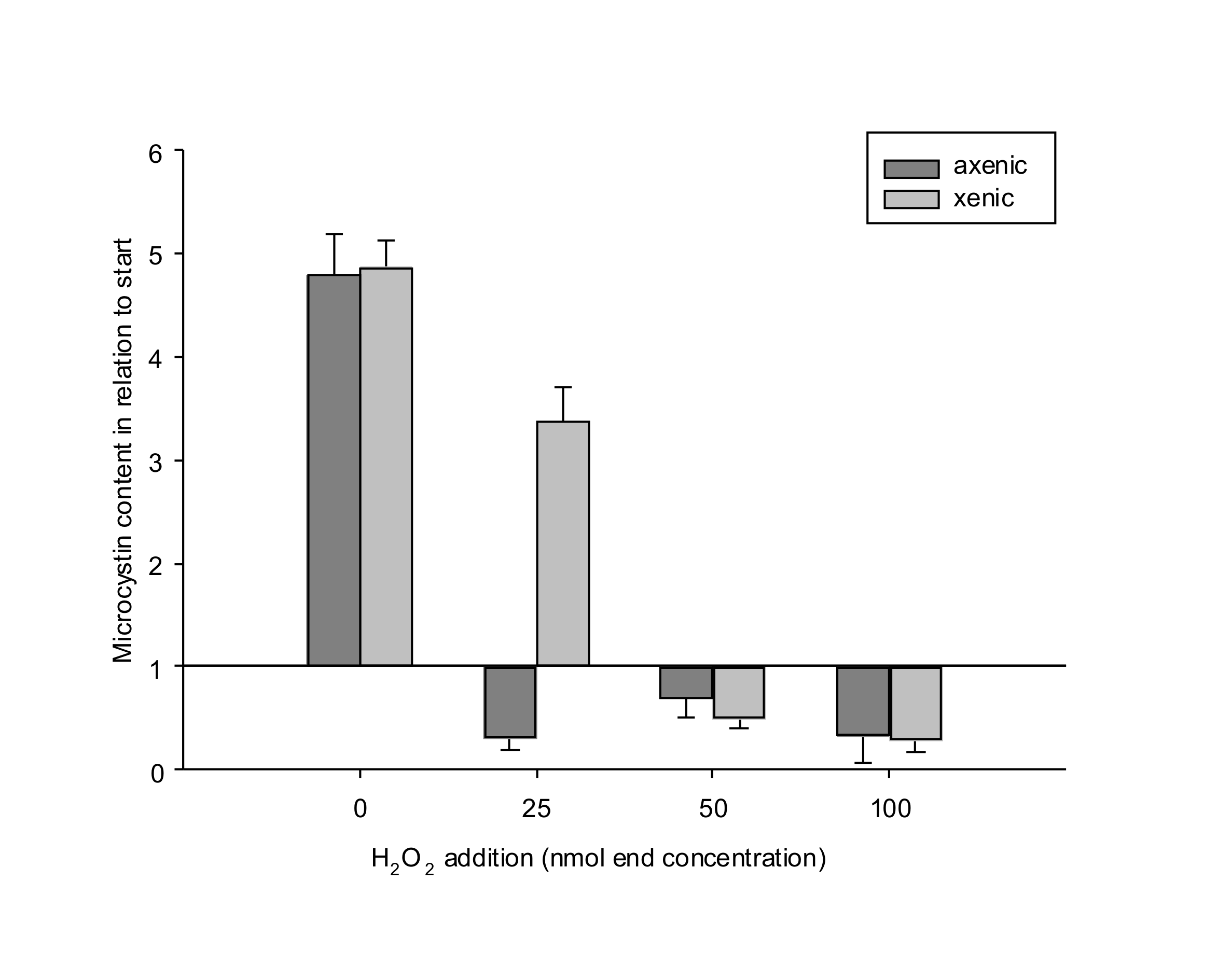

Supplement: Figure S2 — Microcystin content of M. aeruginosa after addition of H2O2. Microcystin content in relation to experimental start of the strain PCC 7806 without (axenic) or with (xenic) accompanying bacteria. (TIF) [file pone.0025569.s002.tif]
